# Advances in Kaempferol: Extraction, Biosynthesis, and Application with Antibacterial Agents

**DOI:** 10.3390/antibiotics14121254

**Published:** 2025-12-11

**Authors:** Xiaojuan Wei, Weiwei Wang, Rongbin Hu, Xun Gao, Bing Li, Yubin Bai, Jiyu Zhang

**Affiliations:** 1 Lanzhou Institute of Husbandry and Pharmaceutical Sciences, Chinese Academy of Agricultural Sciences, Lanzhou 730050, China; 2 Key Laboratory of Animal Drug Production in Ministry of Agriculture and Rural Affairs, Lanzhou 730050, China; 3 Key Laboratory of New Veterinary Medicine Engineering in Gansu Province, Lanzhou 730050, China

**Keywords:** kaempferol, biosynthesis, antibacterial, synergy, nanoparticles

## Abstract

The spread of multidrug-resistant bacteria around the world is rising, and new antibiotics are urgently needed to address the drug resistance crisis. Natural products have become a focal point in the research and development of antibacterial drugs, owing to their structural diversity and biological activity. Kaempferol, in particular, exhibits a range of biological properties, including antioxidant, antibacterial, and antitumor activities, and holds potential for application across various domains. This review aims to provide a comprehensive overview of the extraction, biosynthesis, and antibacterial activity of kaempferol, as well as to elucidate its antibacterial mechanisms. In addition, we also reviewed the synergistic combination of kaempferol with antibiotics, such as the combination of kaempferol with colistin or penicillin, which significantly improved their therapeutic effect. Finally, the preparation of kaempferol nanoparticles and their applications in antibacterial treatments were discussed.

## 1. Introduction

The proliferation of superbugs and multidrug-resistant pathogens has become a significant challenge to global public health [1]. Reports indicate that by 2050, antimicrobial resistance in bacteria could lead to about 10 million deaths each year [2]. There has been a notable reduction in the development and approval of new antimicrobial agents over the past few decades. To address the antibiotic resistance crisis, there is a pressing demand for innovative antibiotics. Most pharmaceuticals on the market are derived from natural products, which are celebrated for their structural diversity and complexity [3,4,5]. Natural products and their derivatives are estimated to constitute 38% of the drugs approved by the FDA [6].

**Figure 1:**
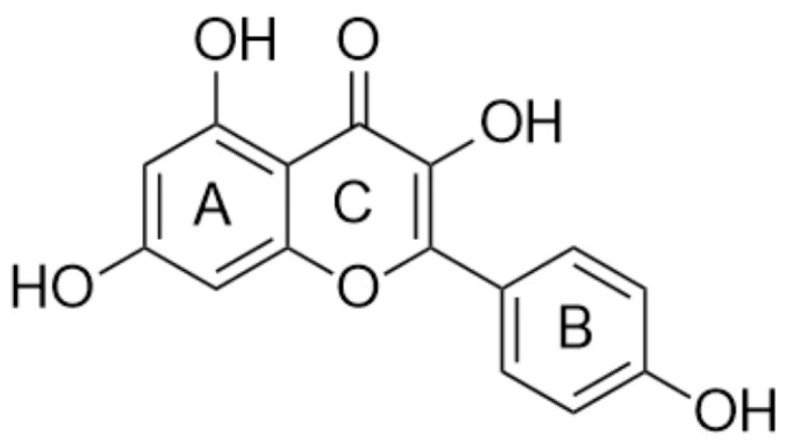
Chemical structure of kaempferol.

Kaempferol is extensively found in numerous fruits, vegetables, tea, and herbs [7]. This compound is chemically identified as 3,5,7-trihydroxy-2-(4-hydroxyphenyl)-4H-1-benzopyran-4-one [8]. The core structure of kaempferol is 2-phenylchromone (C6-C3-C6) [9], its molecular formula is C_15_H_10_O_6_, and its chemical structure is shown in Figure 1. This compound has attracted interest from pharmacologists, medicinal chemists, and dieticians due to its diverse biological activities, including antioxidant, antiaging, anti-inflammatory, antidiabetic, anticancer, anti-obesity, anti-asthmatic, antiplatelet, antiallergic, cardioprotective, and bone-health-promoting properties [10,11]. Kaempferol represents a market value exceeding 5.7 billion $ in the international pharmaceutical and food sectors [12]. With the deepening of research, more and more pharmacological activities and underlying mechanisms are anticipated to be elucidated. This article reviews the application of kaempferol in terms of its antibacterial effects.

## 2. Sources of Kaempferol

### 2.1. Extraction from Plants, Fruits, and Other Species

#### 2.1.1. Conventional Organic Solvent Extraction

Kaempferol is ubiquitously distributed across a variety of plant species, herbs, fruits, sweets, wine, etc. (Table 1). It is isolated and identified using organic solvent, such as methanol, ethanol, petrol, and methanol–dichloromethane. The yield of kaempferol ranges from 0.1 μg/g to 2591.9 μg/g. Pure kaempferol can be isolated from the following species (Table 1).

#### 2.1.2. Supercritical Fluid Extraction (SFE)

SFE uses supercritical fluids as extraction solvents. In this method, CO_2_ is a typical supercritical solvent with no toxicity or adverse effects. Using SFE, kaempferol was isolated from *P. lactiflora* seed oils, with concentrations up to 98.95 ± 4.18 μg/g (dry basis) in samples [188]. Liza et al. isolated kaempferol from *Strobilanthes crispus* leaves by using SFE. Under the optimal conditions, the concentration of kaempferol was 19,450 μg/g in *Strobilanthes crispus* leaves, 98.95 μg/g in *Paeonia lactiflora Pall* seed oils, 110 μg/g in propolis, and 1.96 μg/g in *Ribes nigrum* L. seeds, respectively [189,190,191,192]. Wang et al. isolated kaempferol from *Ampelopsis grossedentata* stems using SFE. With extraction at 25 MPa and 40 °C for 50 min, the yield was highest [193]. A new method using snailase-assisted SFE was devised to extract flavonoids from apple pomace. Under optimal conditions (0.25% snail enzyme, 2 h of enzymolysis, 20% methanol), the concentration of kaempferol reached 0.68 μmol/g [194].

#### 2.1.3. Molecular Imprinting Technique (MIT)

The MIT is a versatile approach designed to target specific molecules by using tailored molecularly imprinted polymers (MIPs) [195].

An active sensing material for the quick detection of kaempferol was developed using a one-pot surface-imprinting synthesis technique. This sensor includes molecularly imprinted polymers (MIPs) that are embedded with carbon dots (CDs) and incorporated into mesoporous molecular sieves (SBA-15). MIPs were successful in detecting kaempferol in vegetables and fruits, with recovery rates of 80% to 112% and a detection limit of 14 μg/L [196]. Cheng et al. used magnetic molecularly imprinted polymers (Fe_3_O_4_ @ SiO_2_-MIPs) to extract and detect kaempferol in apple samples, achieving a recovery rate of 90.5%–95.4% [197].

Novel photo-responsive hollow SnO_2_ molecularly imprinted polymers (PHSMIPs) were developed to extract kaempferol from sea buckthorn leaves [198]. PHSMIPs were created using free radical polymerization, employing kaempferol as the template, 2-(N-3-Sulfopropyl-N, N-dimethyl ammonium) ethyl methacrylate as the photo-responsive monomer, and hollow SnO_2_ as the support, achieving a recovery rate over 90%.

Nanoparticles made from a molecularly imprinted polymer, utilizing a deep eutectic solvent and hexagonal boron nitride (H-BN-MIP), were created for extracting flavonoids from *Ginkgo biloba* leaves through solid-phase extraction, achieving a 94.3% recovery rate [199].

#### 2.1.4. Ultrasound-Assisted Extraction (UE)

UE is a novel technique used to obtain bioactive compounds from plant materials. Using UE, kaempferol was obtained from propolis and bee bread; the yields of kaempferol were 3590 μg/g and 197.60 μg/g, respectively [191,200]. Wójciak, M. *et al*. reported that the content of kaempferol reached 584 µg/g in *Hamamelis virginiana* bark extract using UE. [201]

#### 2.1.5. Microwave-Assisted Extraction (MAE)

MAE is a technology used to extract soluble compounds by microwave radiation. At optimal conditions (90.5% ethanol, 18.6 W/mL microwave power), the yield of kaempferol from *Senna alata* (L.) Roxb was 8540 µg/g [202]. To evaluate the biological activities of *Melaleuca quinquenervia* leaf, the MAE technique was applied with a 20 mL/g liquid–solid ratio and a microwave exposure time of 180 s, obtaining 16,300 µg/g kaempferol [203].

### 2.2. Biosynthesis

Combinatorial metabolic engineering has been explored as an alternative approach for producing flavonoids *in vivo* [204].

#### 2.2.1. Biosynthesis of Kaempferol in Plants

In plants, biosynthesis of kaempferol occurs via the phenylpropanoid pathway. Kaempferol, characterized by a diphenyl propane conformation (C6-C3-C6), is synthesized through the enzymatic condensation of diphenylpropane [10,205]. Phenylalanine serves as a precursor and is oxidatively deaminated by phenylalanine ammonia lyase (PAL) to produce trans-cinnamic acid. Cinnamic acid 4-hydroxylase (C4H) catalyzes the hydroxylation of cinnamic acid to form 4-coumaric acid [206]. 4-Coumaroyl-CoA is formed through activation by 4-coumaroyl-CoA ligase (4CL). Chalcone synthase (CHS) then catalyzes the condensation of this compound with three malonyl-CoA units to synthesize naringenin chalcone. Chalcone isomerase (CHI) converts the intermediate into naringenin through cyclization. Flavanone 3-hydroxylase (F3H) hydroxylates naringenin at C3 to form dihydrokaempferol. Flavanol synthase (FLS) subsequently introduces a C2-C3 double bond to yield kaempferol [206,207].

Producing flavonoids from plants at a large scale is challenging due to extended growth periods, particular cultivation requirements, and their low natural presence. Furthermore, the extraction and purification processes raise expenses [208,209].

#### 2.2.2. Microbial Synthesis

Microbial cell factories that are genetically modified, such as *Saccharomyces cerevisiae* (*S. cerevisiae*) and *Escherichia coli* (*E. coli*), present a promising approach for synthesizing kaempferol [210].

##### Microbial Synthesis in *E. coli*

Peng Xu et al. have successfully engineered an *E. coli* recombinant strain by increasing the level of intracellular malonyl-CoA levels; their naringenin production was 474 mg/L [211]. By optimizing relevant genes and enhancing the intracellular acetyl-CoA pool, recombinant strains were developed, achieving pinocembrin and naringenin production levels of 391 mg/L [212].

##### Microbial Synthesis in *S. cerevisiae*

Duan et al. successfully achieved efficient production of kaempferol (66.29 mg/L in 40 h) in *S. cerevisiae* by introducing a potent flavonol synthase, enhancing acetyl-CoA synthesis, overexpressing the acetyl-CoA biosynthetic pathway, supplementing with p-coumaric acid, and optimizing fermentation conditions [210]. Similarly, *S. cerevisiae* was employed as a host for metabolic engineering to alter its metabolic pathway, facilitating the production of the flavonoid kaempferol from glucose, with a yield of 26.57 mg/L [213].

A recombinant strain with an efficient kaempferol biosynthetic pathway was constructed through screening of essential enzyme genes, engineering an enzyme for synthetic fusion, and increasing gene copy numbers. Through the optimization of fermentation conditions, kaempferol production achieved a yield of 1184.2 mg/L [204].

A cell factory with increased kaempferol production was constructed by metabolically engineering *S. cerevisiae* using a variety of techniques, such as gene screening, phenylethanol biosynthesis branch elimination, core flavonoid synthetic pathway optimization, precursor PEP/E4P supplementation, and mitochondrial engineering of F3H and FLS. Naringenin and kaempferol were obtained at concentrations of 220 mg/L and 86 mg/L, respectively [206].

Using naringenin as a substrate, PGK1 as a promoter, and CYC1t as a terminator, a kaempferol biosynthesis pathway was engineered in *S. cerevisiae* D452-2, catalyzed by rice flavanone 3-hydroxylase. A yield of 19.13 mg/L of kaempferol was achieved [214].

*Guava* leaf tea was fermented using *Monascus anka* and *S. cerevisiae*, followed by hydrolysis with a composite enzyme containing equal parts of cellulase, β-glucosidase, xylanase, and β-galactosidase. Fermentation and intricate enzymatic hydrolysis increased kaempferol levels by 6.8-fold [215].

A modular DNA assembly tool was developed for kaempferol synthesis in *Phomopsis Liquidambaris* (*P. Liquidambaris*). A shuttle plasmid was constructed utilizing *S. cerevisiae*, *E. coli*, and *P. Liquidambaris.* The refactored route employing DNA assembly improved kaempferol synthesis, reaching a yield of 75.38 mg/L [216].

##### Microbial Synthesis in *Streptomyces albus* (*S. albus*) and *Streptomyces coelicolor* (*S. coelicolor*)

Marín L et al. investigated the de novo biosynthesis of kaempferol in *S. albus* and *S. coelicolor*. First, genes necessary for the production of flavonoids were cloned from several organisms, and codons were optimized in *E. coli*. The genes were then cloned into a shuttle vector with a high copy number that was compatible with *Streptomyces* expression. *Streptomyces* J1074 and M1154 were the transformants of the generated recombinant plasmids, and the successful transformants were screened by cultivating on R5A solid medium. Flavonoids were isolated and examined after varying amounts of flavonoid precursors were introduced and cultivated for either 72 h (liquid culture) or 161 h (solid culture) in the following liquid culture experiment. In *S. albus*-pKF, kaempferol (0.212 μM) and its precursors naringenin and dihydrokaempferol were found [217].

## 3. Antibacterial Activity of Kaempferol

### 3.1. Anti Gram-Negative Bacteria

#### 3.1.1. Antibacterial Activity Against *Microcystis Aeruginosa* (*M. Aeruginosa*)

In eutrophic waters, harmful algal blooms have become more frequent and severe recently [218]. *M. aeruginosa* is a major danger to ecological systems and human health [219]. A thorough analysis of the effects of kaempferol on *M. aeruginosa* and its underlying mechanism was carried out. With a dose of 20 mg/L, kaempferol showed a 96.69% inhibitory effect on *M. aeruginosa* at 96 h. By preventing photosynthesis, rupturing cell membranes, decreasing respiratory rate, and altering enzyme function, kaempferol prevents *M. aeruginosa* from growing [220].

#### 3.1.2. Antibacterial Activity *Against Pseudomonas aeruginosa* (*P. aeruginosa*)

*P. aeruginosa* is challenging to remove due to its ability to create biofilms and its resistance to multiple drugs. Kaempferol could effectively inhibit the growth of *P. aeruginosa in vivo* and *in vitro*; the MIC against *P. aeruginosa* 01 was 32 µg/mL [221], and the inhibition zone against *P. aeruginosa* ATCC9027 was 6.8 mm (antibacterial activity was higher than amikacin with 17 mm) [222]. Kaempferol has therapeutic effectivity on the coinfections caused by *S*. *aureus* and *P*. *aeruginosa in vitro* and *in vivo*. Kaempferol therapized the bacterial coinfection by inhibiting *S. aureus* α-hemolysin [223].

Kaempferol inhibited the growth of P. aeruginosa PAO1 in a dose-dependent manner. At 50 µg/mL concentration induced reduction in pyocyanin production and elastolytic and proteolytic activities in *P. aeruginosa* PAO1 by 50% [224].

The mechanism behind this is that kaempferol inhibits the quorum-sensing system by preventing competitive ligands from connecting to LasR [225]. Meanwhile, it reduces acute lung inflammation and damage brought on by *P. aeruginosa* and increases the survival rate of mice by inhibiting the signaling pathway of GSK3β/JNK/c-Jun and NF-κB [226].

#### 3.1.3. Antibacterial Activity Against *Salmonella typhimurium* (*S. typhimurium*)

Kaempferol demonstrated activity against *S. typhimurium*. The inhibition zone against *S. enteritidis* ATCC13076 was 7.2 mm, which was better than that of amikacin (17 mm) [222]. It plays an antibacterial role by reducing transmembrane potential and oxygen consumption.

#### 3.1.4. Antibacterial Activity Against *Xanthomonas* spp.

Plant diseases are a major threat to global food production [227]. Two stubborn plant bacterial infections are *Xanthomonas oryzae* (*X*. *oryzae*) and *Xanthomonas axonopodis* (*X. axonopodis*) [228]. *In vitro*, kaempferol possessed better antibacterial action against *X*. *oryzae* (EC50 = 15.91 µg/mL) than the positive control, thiodiazole copper (EC50 = 16.79 µg/mL) [229]. *In vivo*, kaempferol showed significant preventive and therapeutic properties against rice bacterial leaf blight, with efficiencies of 55.8% and 42.9%, which were higher than those of thiodiazole copper (39.5% and 38.0%). Kaempferol exerts antibacterial effects by damaging the bacterial cell wall or membrane, altering energy metabolism, disrupting the secretion system, and interfering with quorum sensing.

#### 3.1.5. Antibacterial Activity Against *Porphyromonas gingivalis* (*P. gingivalis*)

Kaempferol displayed excellent antibacterial activity against *P. gingivalis*. The MIC towards ATCC 33277 was 20 µg/mL [45], and 8 µg/mL kaempferol showed 84% antibacterial activity against *P. gingivalis* [230]. Kaempferol ameliorates the inflammatory response by targeting the TLR4/MyD88/NFκB signaling pathway in rats [231].

#### 3.1.6. Antibacterial Activity Against *Vibrio cholerae*

Kaempferol had good activity against *V. cholerae* and no toxicity to lymphocytes [41].

#### 3.1.7. Antibacterial Activity Against *Helicobacter pylori* (*H. pylori*)

*H. pylori* infections are associated with stomach diseases like ulcers, gastritis, and perhaps stomach cancer. Both *in vitro* and *in vivo* studies showed that 0.1 mM kaempferol significantly inhibited the growth of *H. pylori* [53,232,233]. Kaempferol may greatly increase the eradication rate of *H. pylori* by suppressing urease activity and modulating virulence factors [234,235,236,237,238]. Furthermore, antibacterial action is linked to fatty acid metabolism, flagellar assembly, ATP-binding cassette transporters, and energy binding with CagA, Urease, and NikR proteins. More importantly, it did not cause any cytotoxicity to host cells [234,239]. HsrA was a necessary protein for microbial viability. This protein is involved in translation, transcription, energy metabolism, nitrogen metabolism and redox homeostasis. Kaempferol exhibited strong bactericidal activities against *H. pylori* strains by binding to HsrA [240].

The MICs of kaempferol against *H. pylori* were 8 µg/mL (ATCC 700392), 16 µg/mL (metronidazole-resistant strain ATCC 43504), and 16 µg/mL (clarithromycin-resistant strain ATCC 700684), respectively [240]. *In vivo*, 5.0 mg/body of kaempferol decreased *H. pylori* levels significantly [53].

#### 3.1.8. Antibacterial Activity Against *E. coli*

Kaempferol demonstrated significant antibacterial activity against *E. coli* [241,242,243,244]. The MICs were 12.5 µg/mL, 128 µg/mL (ATTC 10536), 64 µg/mL (AG102), 25 µg/mL (ATCC 25922), and 64 µg/mL (ATCC 8739), respectively [221,245,246,247]. The inhibition zone for *E. coli* 25922 was 7.2 mm, which was better than amikacin (17 mm) [222].

An important mechanism of kaempferol against *E. coli* is inhibition of DNA gyrase. Kaempferol’s inhibitory activity against DNA gyrase is caused by the obligatory C-4 keto group and hydroxyl group substitutions at C-3, C-7, and C-4′. For flavonols, 4′-OH increased antibacterial activity, while 3′-OH and 5′-OH decreased activity [245].

#### 3.1.9. Antibacterial Activity Against Acinetobacter baumannii

Kaempferol effectively prevented biofilm formation and reduced the number of mature biofilms of *A. baumannii* [248,249].

#### 3.1.10. Antibacterial Activity Against *Klebsiella pneumoniae* (*K. pneumoniae*)

Kaempferol exhibited antibacterial activity against *K. pneumoniae*. The MICs were 64 µg/mL (multidrug-resistant MDR-KP5), 128 µg/mL (ATCC 11296), and 64 µg/mL (KP55, ATCC 11296), respectively [245,246,250].

A dose of 10 μM kaempferol can inhibit growth of *K. pneumoniae*. The mechanism is that kaempferol inhibits dNTP binding of the primary replicative DnaB helicase of *K. pneumoniae*, specifically disrupting the DnaB-dATP interaction. DnaB is an essential component for bacterial survival [251]. Moreover, kaempferol exerts antibacterial effects by disrupting biofilm integrity and enhancing cell membrane permeability.

Kaempferol plays an important role in the PI3K/AKT and MAPK signaling pathways. It mainly binds to the active site of PI3K through hydrogen bonds and salt bridges with binding energies of −6.677 kcal/mol; it binds to AKT1 and MAPK1 through hydrogen bonds, with binding energies of −6.597 kcal/mol and −7.090 kcal/mol, respectively [252]. The impact of kaempferol on *K. pneumoniae* biofilms was moderate to weak.

#### 3.1.11. Antibacterial Activity Against *Proteus mirabilis*

Kaempferol exhibited efficacy against *P. mirabilis* [253].

### 3.2. Anti Gram-Positive Bacteria

#### 3.2.1. Antibacterial Activity Against *Streptococcus pneumoniae* (*S. pneumoniae*)

*S. pneumoniae* triggers pneumonia, otitis media, septicemia, and meningitis, and is the most common pathogen of community-acquired pneumonia (CAP). [254]. *S. pneumoniae* secretes virulence factors of capsule, pneumolysin (PLY), and sortase A (SrtA); these virulence factors are essential for bacterial colonization and spread [255,256,257]. The MIC of kaempferol against *S. pneumoniae* was 12.50 µg/mL [247].

Kaempferol (8 µg/mL) inhibits SrtA transpeptidase activity and biofilm development and maturation. Furthermore, kaempferol greatly reduced PLY-mediated hemolytic activity in a concentration-dependent manner by disrupting PLY oligomerization and reducing its toxicity. Kaempferol significantly reduced the adherence of *S. pneumoniae* to A549 cells. Kaempferol therapy significantly lowers bacterial load and lung damage in mice. Overall, kaempferol exhibited significant anti-infective effects in both *in vitro* and *in vivo* studies [256]. Kaempferol is an efficient therapy for CAP due to its inhibition of the PI3K/ATK/NF-κB signaling pathway, alleviating CAP-related lung tissue injury [258].

#### 3.2.2. Antibacterial Activity Against *Streptococcus mutans* (*S. mutans*)

Kaempferol exhibited significant antibacterial activity against *S. mutans*, achieving a 97% growth suppression rate at concentration of 8 µg/mL [230]. The acidogenicity and acidurity of *S. mutans* were significantly reduced by 1~4 mg/mL kaempferol [259,260]. The mechanisms behind this include that kaempferol decreases ATPase activity and prevents the formation of biofilms. Using 0.5 mg/mL of kaempferol decreased F-ATPase activity by 47.37% [230,259,260].

#### 3.2.3. Antibacterial Activity Against *Listeria monocytogenes* (*L. monocytogenes*)

*L. monocytogenes* is one of the most significant foodborne bacteria. Public health is seriously threatened by the high death rate (20–30%) of *L. monocytogenes* infection [261]. The pathogenicity of *L. monocytogenes* depends on the pore-forming activity of listeriolysin O (LLO), which is essential for the intracellular lifecycle, barrier permeability, colonization, and transmission of *Listeria* [262].

Researchers discovered that kaempferol is an efficient LLO inhibitor, blocking LLO-mediated membrane perforation and barrier disruption. LLO pore formation was successfully suppressed by kaempferol at 32 µg/mL [262]. In addition, kaempferol inhibits NADH oxidase activity and destroys cell membrane integrity, therefore protecting against *L. monocytogenes* [263]. The MIC of kaempferol against *L. monocytogenes* ATCC 1392 was 125 µg/mL [264].

#### 3.2.4. Antibacterial Activity Against *Staphylococcus aureus* and *Staphylococcus epidermidis*

Kaempferol showed notable antimicrobial activity against *S. aureus* and *S. epidermidis*. The MICs of kaempferol against *S. aureus* were *6.25* µg/mL, 7.8 µg/mL (ATCC 49444), 32 µg/mL (ATCC 25923, FDA 209P), 8 µg/mL (USA300), 16 µg/mL (Mu50), and 8 µg/mL (VRS1, VRS5), respectively [221,247,264,265,266].

Kaempferol reduced intracellular colonization of *S. aureus* in lung epithelial cells by about 80%; moreover, its intracellular antibacterial activities surpassed the extracellular activity. The mechanism behind this is that kaempferol mitigates membrane damage and inhibits apoptotic cell death by internalized bacteria [267]. Kaempferol binds to β-lactamase, thereby inhibiting its activity and reducing the secretion of β-lactamase into the external environment [268].

#### 3.2.5. Antibacterial Activity Against *Mycobacterium tuberculosis* (*M. tuberculosis*)

A total of 10.8 million new cases of tuberculosis and 1.25 million fatalities worldwide were attributed to *M. tuberculosis* in 2023 [269]. Standard medications for tuberculosis are no longer effective due to the emergence of multidrug-resistant bacteria [270]. CYP121 enzyme is essential for *M. tuberculosis* to survive [271]. Kaempferol is the most effective CYP121 inhibitor, with inhibition primarily stabilized in the binding pocket by hydrogen bonds and hydrophobic interactions [91].

1-deoxy-d-xylulose-5-phosphate reductoisomerase (DXR) is a key enzyme in *M. tuberculosis*. Kaempferol stably binds at the active site of DXR. Additionally, its good lipophilicity raises the probability of uptake by the *M. tuberculosis* cell wall [272].

#### 3.2.6. Antibacterial Activity Against Enterococcus

Kaempferol exhibited good activity against *E. faecalis,* with MIC of 16 ug/mL (ATCC 29212) [218]. The MIC of kaempferol against *E. hirae* ATCC 10541 was 250 µg/mL [264].

#### 3.2.7. Antibacterial Activity Against *Bacillus cereus* (*B. cereus*)

Kaempferol showed antimicrobial activity against *B. cereus* [241,273]. The inhibition zone against *B. cereus* (ATCC 14579) was 5.9 mm, while that of amikacin was >18 mm [222]. It inhibited *B. cereus* by disrupting cell wall and cell membrane integrity [274].

#### 3.2.8. Antibacterial Activity Against *Bacillus subtilis* (*B. subtilis*)

Kaempferitrin was active against *B. subtilis*, with an MIC of 8 µg/mL [273]; the inhibition zone for *B. subtilis* (*IMG22*) was 6.4 mm, higher than that of amikacin [222].

#### 3.2.9. Antibacterial Activity Against *Micrococcus luteus* (*M. luteus*)

Kaempferol extracted from *Labisa pumila* Benth exhibited moderate antibacterial activities against *M. luteus* [273].

#### 3.2.10. Antibacterial Activity Against *Propionibacterium acnes* (*P. acnes*)

Kaempferol isolated from *Impatiens balsamina* was found to be effective against *P. acnes* [275].

### 3.3. Antifungal Activity

#### 3.3.1. Against *Candida parapsilosis* (*C. parapsilosis*)

Rocha et al. evaluated the susceptibility of kaempferol to *C. parapsilosis* (*C. parapsilosis sensu stricto*, *C. orthopsilosis*, and *C. metapsilosis*). Their results showed that the MIC range was 32–128 µg/mL [276]. The mechanism behind this was that kaempferol inhibited microbial biofilm formation and growth by weakening the cellular adhesion to abiotic surfaces.

#### 3.3.2. Against *Candida albicans* (*C. albicans*)

Kaempferol had good activity against *C. albicans*, inhibiting the growth of *candida* and adhesion to cells. It markedly reduced the number of isolated viable *C. albicans* cells and mortality, and decreased both the metabolic activity and biomass of *C. parapsilosis* biofilms [241,276,277]. The MIC of kaempferol was 12.50 µg/mL [247].

## 4. Synergistic Antibacterial Activity

### 4.1. Combination with Fluoroquinolones Against S. aureus

The combination of tiliroside with norfloxacin, ciprofloxacin, lomefloxacin, and ofloxacin resulted in reductions in the MIC against *S. aureus* by 16-fold, 16-fold, 4-fold, and 2-fold, respectively [278].

A kaempferol-derivative combination with norfloxacin and ciprofloxacin had a synergistic antibacterial effect against MRSA [279]. The combination with norfloxacin reduced the MIC values from 128 µg/mL to 0.25 µg/mL.

### 4.2. Combination with Colistin Against Gram-Negative Bacteria

Polymyxin serves as the final antibiotic option for treating multidrug-resistant Gram-negative bacterial infections. The rise in drug resistance necessitates novel strategies to enhance colistin efficacy, with combining traditional antibiotics and non-antibacterial drugs emerging as a swift and effective treatment option.

The antibacterial effects of combining kaempferol with colistin on colistin-resistant Gram-negative bacteria (*P. aeruginosa*, *E. coli*, *K. pneumoniae*, and *A. baumannii*) were evaluated. The colistin/kaempferol combination exhibited a strong synergistic effect on 83% of strains and additive effects on 17% of strains. Furthermore, the combination showed significant therapeutic effects in both *in vitro* and *in vivo* models, without cytotoxicity [280]. When combined with colistin, kaempferol killed bacteria by inducing dysregulation of iron homeostasis, leading to a lethal build-up of toxic reactive oxygen species [281].

### 4.3. Combination with Penicillin Against S. aureus

Combination of kaempferol and penicillin enhanced sensitivity of *S. aureus* to penicillin G and synergistically inhibited *S. aureus* growth. This was primarily attributed to the downregulation of penicillinase expression and various virulence factors associated with biofilm formation. SarA and/or σB are involved in biofilm development in both the initial and mature stages, and SarA was a potential pharmacological target [282].

### 4.4. Combination with Azithromycin Against S. aureus

*S. aureus* infection may result in osteomyelitis. According to Lei G. et al., the combination of azithromycin and kaempferol significantly inhibited bacterial growth and bone infection. The combined treatment had anti-biofilm activity, reduced oxidative stress, inhibited the phosphorylation of ERK1/2 and SAPK, and further attenuated *S. aureus*-induced osteomyelitis in rats [283].

### 4.5. Combination with Fluconazole Against Candida albicans (C. albicans)

The combination of kaempferol and fluconazole showed a significantly synergistic effect. The MIC_90_ values of fluconazole to different *C. albicans* strains were reduced 2–128-fold. Kaempferol combined with fluconazole exhibits antifungal effects by downregulating CDR1, CDR2, and MDR1 gene expression, thereby decreasing multidrug efflux pump activity and enhancing accumulation [284].

### 4.6. Combination with Aminoglycosides

The combination of a kaempferol derivative with gentamicin presented synergistic effects against *S. aureus* and *E. coli* with MIC reduced 4-fold and 2-fold, respectively. The MICs against *S. aureus* and *E. coli* reduced 4-fold when combined with amikacin [285].

### 4.7. Combination with Ceftiofur

The combination of ceftiofur and kaempferol demonstrated a synergistic antibacterial effect against extended-spectrum-beta-lactamase (ESBL)-producing *E. coli* both *in vitro* and *in vivo* [286]. Kaempferol restored ceftiofur activity on ESBL *E. coli* by influencing β-lactamase activity, biofilm formation, and the AI-2 quorum-sensing system.

### 4.8. Combination with Clindamycin

Kaempferol combination with clindamycin exhibited a greater synergic inhibition of *P. acnes* growth, reducing the MIC 8-fold [275].

## 5. Antibacterial Effect of Kaempferol Nanoagent

To address drug-resistance problems, nanotechnology offers significant potential for the diagnosis, treatment, and prevention of infectious diseases [287].

### 5.1. Lecithin/Chitosan Nanoparticles with Kaempferol Against Fusarium oxysporium

Flavonoid compounds are limited due to their poor solubility and bioavailability. Using the electrostatic self-assembly technique, kaempferol has been successfully encapsulated into lecithin/chitosan nanoparticles (KAE-LC NPs). KAE-LC NPs exhibited inhibition efficacy against *F. oxysporium*, and the inhibition rate reached 67% [288].

### 5.2. Silver Nanoparticles–Kaempferol (AgNP-K) Against S. aureus

Silver nanoparticles incorporating kaempferol (AgNP-Ks) were prepared by a green synthesis method, and its antibacterial activity against MRSA was evaluated [289]. The results demonstrated that AgNP-Ks effectively inhibit MRSA, with an MIC of 1.25 mg/mL. The mechanism behind this is that AgNPs may enhance kaempferol’s effectiveness by increasing bacterial cell wall permeability, facilitating its penetration.

Kaempferol–chitosan/silver (K-CS/Ag) showed concentration-dependent cytotoxicity against human breast cancer cells MDA-MB-231 [290]. A dose of 200 μg/mL K-CS/Ag reduced cancerous cell viability by 91.2% [290]. *Moringa oleifera* extract-loaded silver nanoparticles (Mo-AgNPs) can promote plant growth at 1 µg/mL, 5 µg/mL, and 10 µg/mL. While Mo-AgNPs exhibited toxicity to *Artemia nauplii* at 10 μg/mL, after exposure for 24 h, the mortality rate increased 80% [291].

### 5.3. Chitosan/Sodium Tripolyphosphate Nanoparticles–Kaempferol Against S. aureus

Kaempferol-loaded nanoparticles were synthesized using chitosan and sodium tripolyphosphate via an ion gel method based on electrostatic self-assembly, and subsequently characterized. The anti-quorum-sensing activity of kaempferol-loaded chitosan/tripolyphosphate was evaluated using violacein pigment production in the *C. violaceum* CV026 strain. Loaded chitosan can inhibit quorum-sensing-related processes [292].

### 5.4. Fucoidan-Modified Kaempferol-Loaded Glycyrrhizic Acid Lipid Nanovesicles (Fu-GaLip@KP) Against H. pylori

Fucoidan-modified glycyrrhizic acid lipid nanovesicles containing kaempferol (Fu-GaLip @ KP) were developed, and can effectively eradicate *H. pylori* and restore the diversity of intestinal flora [293]. Kaempferol, encapsulated in glycyrrhetinic-acid-stabilized nanovesicles, effectively penetrates the mucus barrier, disperses biofilms, and eliminates bacteria.

### 5.5. Flavonol-Loaded Cationic Gold Nanoparticles Against S. aureus and E. coli

Li X et al. [294] successfully prepared (11-mercaptoundecyl)-*N*,*N*,*N*-trimethylammonium bromide gold nanoparticles coated with flavonols (flavonol-MUTAB-AuNPs). Flavonol-MUTAB-AuNPs significantly enhanced the inhibition effect on *S. aureus* and *E. coli.*

Kaempferol–gold nanoclusters (K-AuNCs) were non-toxicity to the normal human cell and higher toxicity to the A549 lunch cancer cell [295]. Compared to rose extract, gold nanoparticles (AuNPs) significantly reduced the cytotoxicity on the SH-SY5Y cell line and C6 cell line [296].

### 5.6. Polyhydroxybutyrate–Chitosan–Kaempferol Nanocrystals (PHB-Cs-KAE-NCs) Against S. aureus and A. baumanni

PHB-Cs-KAE-NCs were developed, with powerful antibacterial effects against multidrug-resistant *S. aureus* and *A. baumanni*. *In vitro*, bacterial strain cell viability was reduced by almost 100% after 48 h [297].

## 6. Structure–Activity Relationship of Kaempferol

The structure–activity relationship analysis suggests that, for a good inhibitory effect, hydroxyl group substitution in the A ring in the B ring and the methoxyl group substitution in the A ring are essential. Kaempferol has two hydroxyl groups in the A ring and one hydroxyl group in the C ring. The hydroxyl groups at various places on rings A, B, and C form H-bonds with the important residues of the target, which helps the compounds adhere to the target, thus showing high activity [245,298].

## 7. Conclusions and Prospective

This paper focused on kaempferol, assessing its anti-bacterial properties. Kaempferol exists abundantly in various plants, fruits, and foods. It exhibits widely antimicrobial properties against Gram-negative bacteria, Gram-positive bacteria, and fungi, and holds significant promise as an antibacterial agent *in vitro*. However, its poor solubility and the low bioavailability still led to limited application. A new and effective drug delivery system or preparation of kaempferol analogs are needed. With the development of drug delivery, nanotechnology can modify the monomer of kaempferol, improve its water solubility, increase its bioavailability, and reduce its adverse effects. The development of kaempferol nanoformulations is a great strategy to alleviate antibiotic resistance. However, it is crucial to realize the challenges concerning dosage, stability, antibacterial activity *in vivo*, and routes of administration.

A drug delivery system based on platelet-derived extracellular vesicles (PEVs) was studied, with PEVs loaded with kaempferol (KM) for the treatment of corneal neovascularization. The effectiveness of the PEV-KMs was verified *in vitro* and *in vivo*. The results showed that PEVs had advantages such as biocompatibility and targeting. Meanwhile, the problems of KM hydrophobicity and low bioavailability were overcome [299].

Future research should focus on analyzing antimicrobial mechanisms, exploring intelligent delivery systems to achieve targeted drug delivery in infectious microenvironments, evaluating optimal dosage, and conducting clinical trials to establish pharmacokinetics and toxicity *in vivo*, achieving a thorough understanding of pharmacokinetics, toxicity, stability, safety, efficacy, and mechanisms.

## Figures and Tables

**Table 1:** Species containing kaempferol.

Species	References
*Maytenus ilicifolia* Mart	[13]
*Nigella sativa*	[14,15]
*Sorbus*	[16]
*Sea buckthorn pomace*	[17]
*Opuntia dillenii*	[18]
*Polygonum viviparum* L.	[19]
*Easter lily*	[20]
*Ophioglossum petiolatum*	[21]
*Dennstaedtia scabra*	[22]
*Heterotheca inuloides*	[23]
*Chromolaena moritziana*	[24]
*Ixeridium gracile*	[25]
*Lactuca scariola*	[26]
*Solidago virga-aurea*	[27]
*Helichrysum compactum*	[28]
*Chionanthus retusus*	[29]
*Buddleja indica* Lam.	[30]
*Origanum dictamnus* L.	[31]
*Rosmarinus officinalis*	[32]
*Bupleurum flavum*	[33]
*Echites hirsuta*	[34]
*Cuscuta chinensis*	[35]
*Cuscuta australis*	[36]
*Morinda citrifolia*	[37]
*Morinda morindoides*	[38]
*Vahlia capensis*	[39]
*Solanum nigrum*	[40]
*Combretum erythrophyllum*	[41]
*Cuphea pinetorum*	[42]
*Eucalyptus globulus*	[43]
*Psidium guajava*	[44]
*Syzygium aromaticum*	[45]
*Punica granatum*	[46]
*Pistacia vera*	[47]
*Koelreuteria henryi*	[48]
*Koelreuteria paniculata*	[49]
*Simarouba versicolor*	[50]
*Alternanthera tenella*	[51]
*Oncoba spinosa*	[52]
*Polygonum tinctorium*	[53]
*Thesium chinense*	[54]
*Diospyros lotus.*	[55]
*Pritzelago alpina*	[56]
*Warburgia ugandensis*	[57]
*Warburgia stuhlmannii*	[58]
*Ardisia colorata*	[59]
*Hypericum brasiliense*	[60]
*Vismia laurentii*	[61,62]
Asparagus	[42,63]
Broccoli	[64,65,66,67,68,69,70]
Chinese cabbage (*Brassica rapa*)	[67,69,71,72,73]
Cabbage (*Brassica oleracea*)	[65,69,71,74]
Kale (*Brassica oleracea*)	[65,66,69,75,76,77,78]
Leeks (*Allium ampeloprasum*)	[65,66,68,69,75,79]
Lettuce (*Lactuca sativa var. logifolia*)	[73]
Lettuce (*Lactuca sativa var. crispa*)	[68,71,72,73,74,75,80]
Lettuce (*Lactuca sativa var. capitata*)	[65,67,69,75]
Onions (*Allium cepa or Allium fistulosum*)	[65,66,67,68,69,72,76,77,79,80,81,82,83,84]
Mizuna (Japanese mustard)	[85]
Spinach (*Spinacia oleracea*)	[65,67,68,69,71,74,86]
Tree spinach (*Cnidoscolus aconitifolius*)	[87]
Water spinach	[71,72]
Chives (*Alliumschoenoprasum*)	[75,88,89]
Dill weed (*Anethum graveolens*)	[69,88]
*Foeniculi Fructus*, leaves	[89]
*Europatorium perfoliatum*	[90]
*Pluchea indica*	[91]
*Sambucus nigra*	[92]
*Bunium persicum*	[93]
*Empetrum nigrum* L.	[94]
*Echites hirsuta*	[34]
*Planchonia grandis*	[95]
*Nepenthes gracilis*	[96]
*Rhus verniciflua*	[97]
*Eucalyptus* spp.	[43]
*Tilia tomentosa*	[98]
*Elateriospermum tapos*	[99]
*Euphorbia aleppica*	[100]
*Phyllanthus acidus*	[101]
*Sauropus androgynus*	[102]
*Sebastiania brasiliensis*	[103]
*Populus davidiana*	[104]
*Rhamnus nakaharai*	[104]
*Prunus davidiana*	[105]
*Rosa* spp.	[106]
*Rosa hybrids*	[107]
*Zelkova oregoniana*	[108]
Euonymus alatus	[109]
*Theobroma grandiflorum*	[110]
*Cassia siamea*	[111]
*Indigofera suffruticosa*	[112]
*Indigofera truxillensis*	[112]
*Taxus baccata*	[113]
*Annona cherimola Miller*	[114]
*Allium cepa*	[115]
*aloe vera (Aloe barbadensis)*	[116]
*Lilium longiflorum*	[20]
*Smilax bockii*	[117]
*Dysosma versipellis*	[118]
*Consolida oliveriana*	[119]
*Orostachys japonicus*	[120]
*Rhodiola sachalinensis*	[121]
*Kalanchoe fedtschenkoi*	[122]
*Parthenocissus tricuspidata*	[123]
*Cayratia trifolia Linn*	[124]
*Gynostemma cardiospermum*	[125]
*Tylosema esculentum*	[126]
*Bauhinia vahlii*	[127]
*Acacia nilotica*	[128]
*Amburana cearensis*	[129]
*Cassia angustifolia*	[130]
*Oxytropis falcate*	[131]
*Tadehagi triquetrum*	[132]
*Trifolium alexandrinum*	[133]
*Althaea rosea*	[134]
*Helianthemum glomeratum*	[135]
*Geranium carolinianum*	[136]
*Geranium ibericum subs. jubatum*	[137]
*Pelargonium quercifolium*	[138]
*Brassica rapa*	[139]
*Bunias orientalis*	[140]
*Diplotaxis erucoides*	[140]
*Diplotaxis tenuifolia*	[140]
Broad bean pod, raw	[141]
Common bean [white], whole, raw	[142]
Almond	[143]
Cumin	[144]
Cloves	[144]
Caraway	[144]
Capers	[145]
Lingonberry	[146]
Blueberry	[147]
Cherry	[147]
Cranberry	[147]
Apricots (*Prunus armeniaca*)	[65,68,148,149,150]
Bananas, raw (*Musa acuminata Colla*)	[68,72,151]
Bilberry, raw	[151,152,153,154,155]
Blueberries (*Vaccinium* spp.)	[67,155]
Cashew apple	[156]
Cherries (*Prunus avium*)	[65,68,72,151,153,157]
Chokeberry	[153,158]
Cranberries (*Vaccinium macrocarpon*)	[149,159,160]
Cranberry sauce	[67]
Currants	[65,66,151,153,158,161,162]
Elderberries (*Sambucuss*)	[163]
Goji berry	[164]
Gooseberries (*Ribes* spp.)	[151,158]
Grapefruit (*Citrus paradisi*)	[66]
Grapes (*Vitis vinifera*)	[65,68,151,165]
Juice, sour cherry	[157]
Juice, cranberry cocktail, bottled	[25,67,166]
Juice, grape,	[67,167]
Lemons (*Citrus limon*)	[68,151]
Lingonberries	[66,146,158]
Mangos (*Mangifera indica*)	[67,72]
Melons (*Cucumis melo*)	[68,84,151]
Nectarines (*Prunus persica var. nucipersica*) tina	[68]
Oranges (*Citrus sinensis*)	[67,68,74,151,156]
Papayas (*Carica papaya*	[67,72]
Pitanga (*Eugenia uniflora*)	[156]
Plum (*Prunus domestica*)	[67,68,69,168]
Prickly pears (*Opuntia* spp.)	[169]
Raisins (*Vitis vinifera*)	[67,165]
Red raspberry	[170]
Strawberries (*Fragaria Xananassa*)	[65,66,67,68,74,151,153,156,158,171,172]
Watermelon (*Citrullus lanatus*)	[72,74,151]
Arugula (*Eruca sativa*)	[77,85]
Beans (*Phaseolus vulgaris*)	[65,67,68,74,82]
Brussels sprouts (*Brassica oleracea)*	[59,61,64]
Carrots (*Daucus carota*)	[65,68,69,72,74]
Cauliflower	[64]
Celery (*Apium graveolens*)	[68]
Chicory (*Cichorium intybus*)	[65,80]
Collards (*Brassica oleracea var. viridis*)	[73,76]
Corn poppy	[89]
Cress (*Lepidium sativum*)	[88]
Cucumber (*Cucumis sativus*)	[65,68,69,71,72,74]
Doc (*Rumex* spp.)	[89]
Eggplant	[67,72]
Endive (*Cichorium endivia*)	[65,79]
Garlic (*Allium sativum*)	[68]
Ginger (*Zingiber zerumbet*)	[72]
Hartwort, leaves	[89]
Horseradish	[69,72,75]
Lettuce (*Lactuca sativa var. capitata*)	[75]
*Nelumbo nucifera*	[74]
Lovage, leaves	[88]
Mung beans (*Vigna radiata*)	[74]
Mustard greens (*Brassica juncea*)	[72,76]
Nalta jute	[74]
Pako fern (*Athyrium esculentum*)	[72]
Parsley (*Petroselinum crispum*)	[66,69,74,77,88]
Peas	[67,82]
Peppers (*Capsicum annuum*)	[65,68,69,74,80]
Potato (*Solanum tuberosum*)	[71,74]
Sweet potato (*Ipomoea batatas*)	[67,71,72,76]
Purslane (*Portulaca oleracea*)	[65,76]
Queen Anne’s Lace, leaves	[89]
Radishes (*Raphanus sativus*)	[74]
*Diplotaxis tenuifolia*	[85]
Rutabagas (*Brassica napus var. napobrassica*)	[65,69,76]
Sauerkraut	[65]
Turnip greens (*Brassica rapa*)	[65]
Watercress (*Nasturtium officinale*)	[67,85,88]
Watercress, steamed	[72]
Chia seeds	[160]
Nuts, almonds (*Prunus dulcis*)	[143]
Soybeans, green (*Glycine max*)	[74]
Yardlong bean	[67]
Beans (*Phaseolus vulgaris*, *cv. Zolfino*) (*Phoaseolus vulgaris*, *cv. Zolfino*)	[72,142]
Broad beans (*fava beans*)	[65]
Carob (*Ceratonia siliqua*)	[173]
Cowpeas (*Vigna unguiculata Subsp. Sinensis*)	[174]
Locust bean powder	[173]
Greek greens pie	[89]
Honey, mixed varieties	[175,176,177]
Jams and preserves, apricot	[149,178]
Jams and preserves, grape	[67]
Jams and preserves, guava	[67]
Jams and preserves, peach	[178]
Jams and preserves, raspberry	[179]
Jams and preserves, strawberry	[178,180]
Alcoholic beverages	[94,167,181,182,183,184]
Black tea	[66,70,84,167,185]
White wine	[181,183]
Blackcurrant wine	[67,85,88]
Red wine	[181,182,183,184,186,187,188]

## Data Availability

No data was used for the research described in the article.
